# Myopathy with Concurrent Tadalafil and Simvastatin

**DOI:** 10.1155/2009/378287

**Published:** 2009-08-09

**Authors:** Maria Pia Gargante, Marco Vacante, Cristina Russo, Mariano Malaguarnera

**Affiliations:** Department of Senescence, Urological and Neurological Sciences Ospedale Cannizzaro, Viale Messina, 95125 Catania, Italy

## Abstract

A 48-year-old man, using statin, was admitted to hospital with progressive myalgia after consumption of tadalafil and simvastatin. Muscle pain and penile erection disappeared seven days after interruption of therapy. This case demonstrates the interaction of tadalafil with simvastatin resulting in myopathy. Muscle damage could be attributed to the common metabolic way of these two drugs which is cytochrome P450 isoenzyme system.

## 1. Background

Cardiovascular disease and erectile dysfunction (ED) share similar etiologic and pathophysiologic pathway. Mainly it is well known that most cases of ED derive from an impairment of the vascular endothelium. Recent epidemiological studies have underlined the close association between ED and comorbid conditions such as hypertension, diabetes mellitus, and dyslipidemia. Actually phosphodiesterase type 5 (PDE5) inhibitors are recommended as first-line therapy for erection problems of all etiologies and severities, at the same time statins are a recommended therapy for vascular disorders [1]. Pharmacologic data support a potential interactions between these drugs because PDE5 inhibitors and statins are metabolized by the liver via the cytochrome P450. In this family of enzymes, CYP3A is the dominant CYP in terms of both expression levels in the liver and the number of drugs metabolized [2]. Both simvastatin and tadalafil metabolism involve principally CYP3A. The metabolism of simvastatin in human liver microsomes is catalysed primarily (≥80%) by CYP3A4/5, with a minor contribution (≤20%) from CYP2C8. Tadalafil during its metabolism is converted to a metabolite that binds irreversibly to the enzyme active site, permanently inactivating the enzyme. The inactivated enzyme must be replaced by newly synthesized CYP to regain activity [3]. Today in addition to the classical proerectile-effect, several studies have demonstrated that PDE5 can also influence vascular endothelium; given that the combinated therapy statins plus PDE5 could be very useful in patient with high cardiovascular risk [4, 5].

## 2. Case Report

A 48-year-old man with a history of dyslipidemia and erectile dysfunction was admitted to our department due to a manifestation of progressive myalgia after comsumption of tadalafil. He had been taking simvastatin at doses of 20 mg/die for almost 8 days and recently he was prescribed tadalafil. Approximately 3 hours after the patient began tadalafil, he developed unexplained, severe muscle aches (particularly in the lower extremities). Gradually, these symptoms worsened with severe pain in the 3/5 of surface area over upper extremities and 2/5 of surface area over lower extremities, weakness in the pelvic and shoulder girdle regions. The penile erection was maintained for five days. According to his clinical history we stopped tadalafil and simvastatin therapy and we noted a significant improvement in muscular symptoms after 5 days. Further questioning revealed no symptoms of viral illness, or other identifiable causes of myalgias. He denied unusual or abnormal physical activity, trauma, burns, recent intramuscular injections or symptoms of coronary artery disease. His temperature was 37°C, pulse rate 87 b/min and blood pressure 125/75 mmHg. Laboratoristic analysis are reported in the Table 1. No substantial changes in the ECG were noted. Myopathy, probably drug induced, was diagnosed because there was no evidence of recent trauma or other potential causes of muscle damage. An intravenous fluid supplement was prescribed, but the weakness decreased slightly after he was discharged.

## 3. Discussion

Approximately 95% of statin-treated patients tolerate this treatment without any adverse effects. Tadalafil (Cialis) is a potent reversible phosphodiesterase-5 inhibitor used for the treatment of erectile dysfunction. Physiologically, in response to sexual stimulation, nitric oxide is released into the smooth muscle of the corpus cavernosum of the penis, resulting in elevation of cGMP levels and relaxation of the smooth muscle to produce an erection [6, 7]. Patients who are treated for erectile dysfunction are likely to take additional medications for preexisting conditions such as cardiovascular disease [8]. Statins and tadalafil are both metabolized by the cytochrome P450 isoenzyme system (particularly CYP3A4). In this family of enzymes, CYP3A is the dominant CYP in terms of both expression levels in the liver and the number of drugs metabolized. As a result of the broad substrate specificity exhibited by CYP3A and the polypharmacy often used by patients, alterations in CYP3A activity by induction or inhibition can result in metabolically based drug-drug interactions. In vitro studies were performed examining the ability of tadalafil to alter metabolism mediated by CYP3A in cultures of human hepatocytes or reversibly inhibit metabolism mediated by CYP3A in human liver microsomes. In addition, tadalafil was examined for its ability to cause mechanism-based inhibition of CYP3A-mediated reactions in human liver microsomes. Mechanism-based inhibition occurs when the drug is converted to a metabolite that binds irreversibly to the enzyme active site, permanently inactivating the enzyme. The inactivated enzyme must be replaced by newly synthesized CYP to regain activity; thus recovery is slowed after mechanism-based inactivation as compared with reversible inhibition [2, 3]. Other in vitro studies evaluated the effect of tadalafil on the pharmacokinetics of coadministered drugs that are metabolized by CYP3A such as lovastatin. This study remarks that side effects such as myopathy could be even higher if a statin is used concomitantly with one of the most potent CYP3A4 inhibitors such as tadalafil. So their concomitant use should be avoided. Though the meaning of this case is yet not clear, we speculate that this fatigue may be due to a low-grade muscle damage induced by tadalafil. Muscle toxicity is a potential adverse effect not only for statins, but also with 5PD . So a combination of tadalafil and statins may enhance the effect and induce muscle toxicity [9, 10]. In the patients treated with statins, tadalafil should not be the PDE5 inhibitors of first choice, because of its long half-life in order to avoid potentially life-threatening adverse effects.

## 4. Conclusion

Statins are used to reduce blood cholesterol and have been shown in multiple studies to reduce the risk of heart attacks, heart attack deaths, and strokes. The benefits of treating blood cholesterol with statins have been demonstrated in a wide variety of patient groups, including healthy patients. The major medical problem with statins is that they can produce muscle aching, weakness, and cramps in some patients. The incidence of myopathy could be even higher if simvastatin is used concomitantly with CYP3A4 inhibitors such as 5PDi. Moreover muscle toxicity is a potential adverse effect not only for statins, but also with 5PDi so their concomitant use should be carefully monitored. Specially in the patients treated with statins, tadalafil should not be the PDE5 inhibitors of first choice, because of its long half-life [11]. At the same time the best statin to use might be rosuvastatin because it is not extensively metabolized by isoenzymes of the CYP system [12].

## Figures and Tables

**Table 1:** Serum values of some parameters during 5 days.

Normal range					
AST (14–41 U/L)	65	74	60	51	39
ALT (9–63 U/L)	76	82	71	64	55
*γ* GT (7–50 U/L)	71	96	80	70	72
CK (38–190 U/L)	326	444	384	210	187
Myoglobin (0–116 *μ*g/L)	247	289	204	145	125
Urea (3–8 mmol/L)	16	14	10	8	8
Creatinine (0.06–0.12 mmol/L)	0.15	0.12	0.08	0.06	0.07
Total bilirubin (<20 *μ*mol/L)	15	14	16	14	12
